# New Medicine Service by Community Pharmacists: An Opportunity to Enhance Universal Health Coverage at a Primary Health Level in South Africa

**DOI:** 10.1177/00469580221146834

**Published:** 2023-01-09

**Authors:** Vivian Naidoo, Rajatheran Moodley, Varsha Bangalee, Fatima Suleman

**Affiliations:** 1 University of KwaZulu-Natal, Durban, South Africa; 2 Merebank Durban South Africa, Durban, South Africa

**Keywords:** universal health coverage, new medicine services, South Africa, community pharmacist, primary health care

## Abstract

The implementation of universal health coverage (UHC) in South Africa has focused on promoting equitable health care services to all citizens. In this regard, pharmacists are expected to expand their professional capabilities to promote primary healthcare system functionality. The new medicine service (NMS) has proven to be beneficial in medicine optimization and adherence. The aim of the NMS is to assist and advise patients on their newly diagnosed conditions and to promote the safe and rational use of medicines. This study explores the provision of NMS within the UHC primary healthcare service package and the opportunity for enhancing pharmacist practice. This pilot reports on the implementation of NMS in a low-middle income country. Data was obtained using convenience sampling and an interview-based approach. Findings were evaluated, analyzed, and reported using qualitative techniques. This study was conducted at an independent community pharmacy in Durban, South Africa. Fifty-four patients were successfully enrolled into the program based on the eligibility criteria; 19 patients exited the program before completion. From those that completed the program, 65.71% had no problems detected; rather the program served as a platform to provide information and ensure proper adherence practices, 34.29% of patients experienced problems and were referred back to the prescriber, or pharmacist. After the completion of the program, 54.29% where found to be adherent to their medication, however, 45.71% were found to be non-adherent and were counseled accordingly or referred back to the medical practitioner. This paper highlighted that the implementation of a pharmacist’s full scope of practice and services such as the NMS is essential in improving therapeutic outcomes, recognize medicine related problems, and avert unnecessary use of medicines.

**What do we already know about this topic?**

There is limited literature available on the use of NMS in low-middle income countries though impacts have been recorded in high income countries

**How does your research contribute to the field?**

The utilization of NMS ensures rational and safe use of medicines, reinforces compliances, and can potentially reduce health-related costs. This pilot also assesses the ability to implement NMS in low- and middle-income countries and potential achievements and challenges of this program.

**What are your research implications toward, theory, practice, or policy?**

The introduction of such a service in the NHI service package will enable South African pharmacist to expand their scope of practice and promote patient centered care at a community platform. The pilot study sets up a framework for a larger scaled up study that could also assess the budget implications for set up and the cost savings achieved.

## Introduction

### Universal Health Coverage Implementation in South Africa

Previous health policies have created gaps between the availability and accessibility to healthcare services across the public and private healthcare sectors in South Africa. In an attempt to correct this disparity, the South African government has begun to lay the foundation for new health policies and regulations for transitioning toward Universal Health Coverage (UHC) through the implementation of the National Health Insurance (NHI).^1^ The NHI is a health financing system that was created to pool funds and actively purchase services to provide universal access to affordable and quality healthcare, irrespective of individuals socio-economic status.^1^ Introductory NHI activities focuses on strengthening the health system by improving service delivery. This encompasses the re-engineering of Primary Health Care (PHC) to enhance the performance and quality of health services at public PHC facilities.^1^

The conventional role of a pharmacist is set to change in South Africa as the public-private partnership will enhance services and roles inherent to the scope of practice of a pharmacist that have been previously underutilized in South Africa. The aim of this paper is to review how one such service option, viz. the New Medicine Service (NMS) model may reduce non-adherence, hospital admissions, and progression of chronic conditions.

### New Medicine Service Benefits

Medicine non-compliance particularly for long-term conditions is considered a leading cause of mortality and morbidity in primary healthcare.^2^ Long term treatment regimens are dependent on patients self-care and management, correct medicine use, and lifestyle modification in order to produce positive therapeutic outcomes.^2,3^ Medical reviews across different countries and disease states found that between 30% and 50% of prescribed medicines are not taken as intended.^4^ Non-adherence is a major problem that often goes unrecognized by prescribers and is usually undisclosed by patients.^4^ Adherence can be measured by direct or indirect means; direct measures include a variety of calculations based on drug concentration levels in the body.^5^ However, more commonly used are the indirect measures which comprises of patient questionnaires, structured interviews, clinical responses, refill rates, and pill counts.^5^ A common medical error is the assumption that patients fully understand their medicine regimens and are taking it correctly; however, most often patients require more information and guidance upon initiation by prescribers.^4^ This gap in knowledge emphasizes the need for a pharmacist to work in a capacity that promotes adherence by consulting with patients on a one-on-one basis to create a platform for understanding and producing positive health outcomes, reducing negative health related outcomes as well as medical associated cost. The New Medicine Service (NMS) is an advanced service provided by a pharmacist to support patients, with newly diagnosed conditions, and prescribed medicines, with the aim of improving medicine adherence. It is focused on specific patient groups and conditions and entails 2 office-based consultations with a pharmacist with the requisite competencies. The implementation of the NMS focuses on bridging this gap between patient and clinician by measuring adherence via indirect measures and medicine related problems.^6,7^

The primary aims of the NMS is to aid in advising on the newly diagnosed condition and medicine, alleviating patient anxiety, clarifying treatments prescribed and administration, decreasing the symptoms and long term complications associated with chronic conditions, identify medication taking problems, provision of additional information, and instructions for better self-management and to promote better lifestyle changes.^4^ The NMS focuses on reducing problems that arise with newly prescribed medicines for patients on long-term treatment that may quickly become non-adherent.^8^ Although, all of these abilities fall within the scope of practice of a pharmacist, these are untapped skills as pharmacists in South Africa take on the conventional role of “custodians of medicine.” Currently the majority of patients utilize the outpatient services in public healthcare facilities, hence pharmacists experience a heavy workload of medicine dispensing, distribution, and administration, resulting in a small percentage of pharmacists available to provide clinical pharmacy services.^9^ Therefore, repositioning some community pharmacists to focus on the advanced services will alleviate some pressure on the healthcare system.

The framework of the NMS is centered around a systematic approach that requires a standard operating procedure, obtaining the patient’s consent, supplying patients with information and instructions on the NMS and an interview conducted with patients to initiate and shape their treatment and follow-up stages of the service.^8^ In order to commence the service it is essential to have a registered pharmacist with the appropriate additional training, consultation with and cooperation of prescribers, and a suitable location for conducting interviews.^4^

### International Experiences With the NMS

The United Kingdom (UK) implemented the NMS for patients that present a higher risk of hospitalization. It was found to be beneficial for these patients to be referred to the hospital pharmacist for a comprehensive evaluation to prevent relapse.^10^ The implementation of such a program in the UK has produced many positive health outcomes as well as massive cost-savings.^8^ Findings from other UK based studies exhibited that when the NMS was performed, patients adherence was 10% higher (70.7%) when compared to the control group (60.5%).^11^ Similarly, another study found that the NMS not only increased patient medicine adherence but also translated into significant health gain resulting in reduced overall cost with a 96.7% probability of cost effectiveness when compared to standard practice.^12^ Since then, this service has been implemented on a wide scale with 12 485 UK based pharmacies claiming payment for services offered to patients in their community in October 2019.^13,14^

Furthermore, to substantiate these findings another report by the European Union confirmed that between 2014 and 2015, 774 930 NMS reviews were conducted on target groups (high risk medicines, post-discharge, respiratory, and cardiovascular patients) which resulted in significant cost savings as well as improved patient adherence by 10% (16). In France, this service was nationally commissioned to be conducted for patients that were starting vitamin K antagonist therapy; by June 2013, 230 000 NMS review interviews were conducted and by December 2014, 14 584 pharmacies were performing the service with a patient satisfaction score of 8.7/10.^15^ Since then France has expanded the conditions to which this service may be offered due to the positive health impact it produced.^15^ Similar resolves of improved adherence in chronic conditions with the implementation of an NMS were observed in other European countries such as Belgium and Norway.^15^

Therefore, this model presents a potential for a pharmacist-initiated service for SA to improve medication adherence in patients and reduce the burden and costs of complications to the health system. A model was thus implemented as a pilot study to assess the feasibility in a community pharmacy setting. This was followed by evaluating the impact that pharmacist interventions and assessments had on outcomes related to medicine therapy. The pilot process and findings can then be used to recommend a model framework for the NMS incorporation into NHI.

## Methods: Feasibility of Implementing and Adapting an NMS Model in a Community Pharmacy in South Africa

### Inclusion Criteria

This was a pilot study using convenience sampling of a single independent community pharmacy in Durban, KwaZulu-Natal, South Africa, that services a lower-to middle-income community. Prior to offering the NMS, it was important to establish a need for such a service. According to the dispensing data available in the pharmacy, type-2 diabetes, dyslipidemia, hypertension, and asthma were most prevalent in the community in which the pharmacy was located. These conditions are also key drivers of the non-communicable diseases profile in South Africa (20).

The new medicine service commenced with the patient’s initial presentation with a prescription from a medical practitioner for a newly prescribed medication according to a predetermined list developed by the pharmacist, that treated 1 or more of the 4 long-term conditions (LTCs; Supplemental Table).^16^ Only, patients that were 14 years and older and able to give consent were initiated into the program.

### Setting Up NMS

In order to provide the service, the pharmacy ensured competency of 2 pharmacists by completing the on-line CPD (continuing professional development) offered by the Centre for Pharmacy Postgraduate Education (CPPE) in partnership with the National Health Service (National Health Service in the United Kingdom—NHS-UK) and the University of Manchester (as no such training was available in South Africa). Secondly, the pharmacy established a fully functional office as the private consultation area, together with a computer, filing system, and a telephone line. Thereafter, a communication strategy was established, which included setting up a referral system together with the local medical practitioners through discussions and via official letters from the pharmacy. The study was granted ethical clearance by the University of KwaZulu-Natal, Biomedical Ethics Committee, South Africa (BREC Ref No. BE626/17). Patients were selected via a total sampling technique. Patients were informed about the service and invited for a one-on-one consultation with a pharmacist which usually took an average length of 25 min. The strategy was extended to patients via pamphlets distributed in the pharmacy and in doctors’ rooms and via the pharmacy’s Facebook page. Essential to the process was to establish legal compliance that included documented record keeping.

Patients were notified that their participation in the program was voluntary and that they could withdraw at any point. Every patient filled in a consent form after being fully informed about the service that was provided. Patient interviews were conducted in-person in a private consulting room to maintain confidentiality. Regular debriefing with another pharmacist and reviewing of clinical notes was performed to ensure that bias was minimized, and the pharmacist was actively aware of the line of questioning. Thereafter, based on the consultation with the pharmacist, a patient data form was generated encompassing all the patient’s demographic and medical information as well as follow-up dates. An interview questionnaire, adapted by the pharmacist for this service from the NHS-UK, was used to obtain information which was divided into 4 sections that focused on the patient’s: demographic information, medical history and disease profile, newly prescribed medicines, action plans, and referral were applicable. A qualitative approach was used in the newly prescribed medicine section of the questionnaire to gain a more in depth understanding of each patient’s experience in taking their medicines. Therefore, questions included: “what am I taking this medicine for?”, “when should I be taking my medicines?”, “have I missed any doses?”, “are there any special instructions to follow?”, “am I experiencing any side effects?”. Thereafter, both the pharmacist and the patient came to an agreement on the adherence strategy that needed to be adopted, follow-up appointments, resolutions to any problems, and if a referral back to the prescriber was necessary.

**Figure 1.**
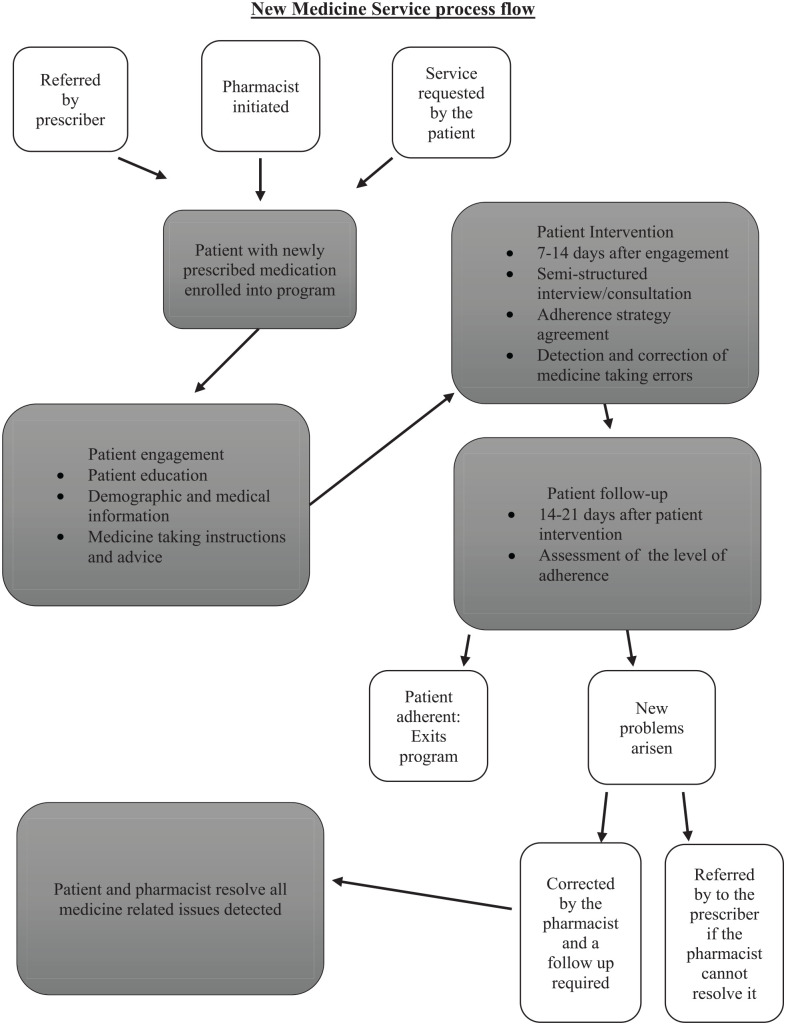
Process of new medicine service implementation.^17^

A second follow-up interview was utilized, between 14 and 21 days after the first interview, to determine the level of adherence as per patient disclosure and to ascertain if any new problems had arisen. At this point the pharmacist chose to either exit the patient from the program due to adequate adherence or implement another follow-up in 7 days should a new problem have arisen, or contact the prescriber if he/she could not resolve it on their own (Figure 1). A patient was defined as non-adherent if any doses were missed without consultation or agreement with the medical practitioner or pharmacist.

### Pharmacy Reported Outcomes From the Pilot

Data was collected and documented over a 6-month period during which no changes were made to the method. Fifty-four patients were successfully enrolled into the program based on the eligibility criteria. A discontinuation criterion was established at this point as the aim of the study was to pilot the NMS. Nineteen patients exited the program before completion. Patients were categorized according to their long-term treatment (some presenting with multiple co-existing conditions); 40% of the patients had type-2 diabetes, 54.29% of patients had hypertension, 17.14% of patients had dyslipidemia, and 5.71% of patients had asthma (Table 1).

**Table 1.** Statistical Findings From Patients Initiated in NMS Program.

Variables	Number of patients (n)	
Patients enrolled into program	n = 54	
Patients that exited without completion	n = 19	
Patients completed program (n = 35)		
Patients that presented with no problems and exited the program after completion	n = 23	65.71%
Patients that presented with problems during the program and were referred back to the prescriber/pharmacist initiated therapy	n = 12	34.29%
Patients that remained adherent after the follow-up	n = 19	54.29%
Patients that were non-adherent after the follow-up	n = 16	45.71%
Long-term conditions that the patients presented with		
	Hypertension	19
	Type 2 diabetes mellitus	14
	Dyslipidemia	6
	Asthma	2

Out of the 35 patients (n = 35) that completed the program, 65.71% (n = 23) had no problems detected by the end; rather the program served as a platform to provide information and ensure proper initiation of new chronic medication. Twelve (34.29%) patients experienced problems and were referred back to the prescriber, or pharmacist intervention was required after follow-up visits. After the completion of the program by all patients, 54.29% (n = 19) where found to be adequately adherent to the newly prescribed medication based on evaluation of their medicine-taking practices, structured interviews, and questionnaire; and exited the program. However, 45.71% (n = 16) were found to be non-adherent after completion of the program and were counseled accordingly or referred back to the medical practitioner.

Any increase in patient engagement with both their disease condition and their medicines contributed positively to their understanding and to their adherence. Early intervention before the first repeat identified patients having difficulty in taking their medicines. Intervention models including pharmacist interventions to mitigate side-effects and the possible need to refer back to the treating prescriber ensured a lower drop-out rate, and identified problems in medicine management in early diagnosis of chronic patients.

### Limitations Reported From the Pilot

As this was a new service, the uptake was slow. Constant reminders had to be sent to the participating medical practitioners in the area and to the dispensing unit in the pharmacy to identify potential patients and refer. The process entailed 2 consultations but due to the drop-out after the first consultation (adequately informed and did not need further pharmacist intervention as per telephonic contact), it was difficult to get all participants to complete both consultations. The process also required a daily allocation of 3 h (2-5 PM) for 1 pharmacist to be office bound to ensure the program was sustained.

## Discussion

The findings of this case study provide evidence for further investigation in the implementation and adaptation of the NMS. The pharmacist detected therapy problems in 22.25% of the patients initiated on chronic medication. The absence of this intervention could have resulted in these patients experiencing problems for a long period of time without detection. Furthermore, 67.71% of patients exited the program with no problems detected indicating that proper initiation of LTC treatment curbs the possibilities of early errors in chronic treatment, that could worsen if not resolved. It further serves as a platform for patient education and reinforcing corrective medicine taking practices.

The service was notably advantageous to patients that had more than 1 LTC as they were on multiple medication regimens and therefore required reinforced information and instructions to promote adherence. This also gave them a platform to discuss any concerns or confusion; and enhanced communication pathways to address any health concerns. Studies of larger sample size and more pharmacies are required to assess the full impact of the model. For such a program to be scalable and sustainable, it requires funding as in most other countries. The system will also need to be converted from a paper based program to a technology driven resource with centralizing of the intervention in order to have a scaled quality measure. The results of our case study can be further reinforced as findings from other studies have exhibited that NMS considerably improved the proportion of patients adhering to their new medicines by 10% when compared with normal practices. Furthermore, it allowed for additional advice and reassurance after the diagnosis and commencement of new treatment.^17-19^

It is important to note that no competency standards or training on this aspect exists in South Africa. The pharmacy used a UK course to attain the necessary competency and training. The South African Pharmacy Council would need to be consulted in terms of developing local standards and competencies for training institutions to offer the course for South African pharmacists. The study did not look at the cost implications of setting up such a service, which is required to guide further implementation and scale-up.

## Conclusion

Medicine utilization interventions like the NMS encapsulates UHC aims surrounding rational medicine use, cost savings, and revitalization of PHC in South Africa. However, the successful implementation of such services is dependent on community pharmacist integration into primary health care, reimbursement, and adequate training of pharmacist. Reviewing a patient’s pharmacotherapy is an integral part of providing care for patients that have long-term conditions to achieve the best possible outcomes. The implementation of the NMS service in this study exhibited the benefit of reinforcing correct medicine taking practice, thereby improving adherence. Furthermore, the service provided a platform to assist patients experiencing difficulty taking their newly prescribed medicines and improve understanding. Future studies, extensive engagement with the pharmacy workforce and medical practitioners, robust piloting, a phase rollout approach, and relevant policy changes in South Africa are essential to ensure that services such as NMS meet their intended aims in practice and should be set a as a national priority.

## Supplemental Material

sj-docx-1-inq-10.1177_00469580221146834 – Supplemental material for New Medicine Service by Community Pharmacists: An Opportunity to Enhance Universal Health Coverage at a Primary Health Level in South Africa Click here for additional data file. Supplemental material, sj-docx-1-inq-10.1177_00469580221146834 for New Medicine Service by Community Pharmacists: An Opportunity to Enhance Universal Health Coverage at a Primary Health Level in South Africa by Vivian Naidoo, Rajatheran Moodley, Varsha Bangalee and Fatima Suleman in INQUIRY: The Journal of Health Care Organization, Provision, and Financing
