# Supplementary material for: New Medicine Service by Community Pharmacists: An Opportunity to Enhance Universal Health Coverage at a Primary Health Level in South Africa
Source: Inquiry. 2023 Jan 9;60:00469580221146834. doi: 10.1177/00469580221146834 (PMC9834920; doi:10.1177/00469580221146834)
Supplement: sj-docx-1-inq-10.1177_00469580221146834 – Supplemental material for New Medicine Service by Community Pharmacists: An Opportunity to Enhance Universal Health Coverage at a Primary Health Level in South Africa [file sj-docx-1-inq-10.1177_00469580221146834.docx]

Supplementary table: Specified medicines for NMS corresponding to the SAMF (16)

| Generic name | SAMF category | SAMF pg no |  | Generic name | SAMF category | SAMF pg no |
| --- | --- | --- | --- | --- | --- | --- |
| Acarbose | A10BF | 77 |  | Lisinopril | C09A | 161 |
| Acebutolol | C07AB | 152 |  | Losartan | C09C | 163 |
| Aminophylline | R03DA | 543 |  | Lovastatin | C10AA | 169 |
| Amlodipine | C08C | 156 |  | Metformin | A10BA | 73 |
| Aspirin | B01AC | 102 |  | Methyldopa | C02AB | 138 |
| Atenolol | C07AB | 152 |  | Metoprolol | CO7AB | 153 |
| Atorvastatin | C10AA | 168 |  | Minoxidil | CO2DC | 141 |
| Beclometasone | R03AB | 538 |  | Moexipril | CO9A | 160 |
| Bezafibrate | C10AB | 172 |  | Mometasone | D07A | 200 |
| Bisoprolol | C07AB | 152 |  | Montlukast | R03DC | 543 |
| Budesonide | R03BA | 539 |  | Moxonidine | C02AC | 139 |
| Candesartan | C09C | 163 |  | Nateglinide | A10BX | 78 |
| Captopril | C09A | 159 |  | Nebivolol | C07A | 153 |
| Carvedilol | C07A | 153 |  | Nifedipine | C08C | 155 |
| Ciclesonide | R03BA | 539 |  | Nimodipine | C08C | 157 |
| Cilazapril | C09A | 160 |  | Perindopril | C09A | 160 |
| Clonidine | N02CA | 438103 |  | Piogliatzone | A10BG | 77 |
| Clopidogrel | B01AC | 105 |  | Prazosin | C02CA | 140 |
| Dabigartran etexilate | B01AE | 105 |  | Pravastatin | C10AA | 169 |
| Diltiazem | C08 | 158 |  | Propranolol | C07AA | 151 |
| Dipyrimadole | B01AC | 103 |  | Quinapril | CO9A | 161 |
| Doxazosin | C02C | 140 |  | Ramipril | CO9A | 161 |
| Enalapril | C09A | 159 |  | Repaglinide | A10B | 78 |
| Ephedrine | N01AH | 417 |  | Rosuvastatin | C10AA | 169 |
| Eprosartan | C09C | 163 |  | Salbutamol | R03AC | 536 |
| Ezetimibe | C10AB | 172 |  | Salmetorol | R03AC | 538 |
| Felodipine | C08CA | 157 |  | Sildenafil | G04BE | 249 |
| Fenoterol | R03AC | 537 |  | Simvastatin | C10AA | 167 |
| Fluticasone | R03BA | 540 |  | Sitagliptin | A10BH | 78 |
| Fluvastatin | C10AA | 168 |  | Sodium cromoglicate | R01AC | 526 |
| Formoterol | R03AC | 539 |  | Sotalol | C01BD | 130 |
| Fosinopril | C09A | 163 |  | Tadalafil | G0ABE | 250 |
| Gemfibrozil | C10AB | 172 |  | Telmisartan | C09C | 164 |
| Gliblenclamide | A10BB | 74 |  | Terazosin | G04CA | 252 |
| Glicazide | A10BB | 75 |  | Terbutaline | R03AC | 537 |
| Glimperide | A10BB | 76 |  | Theophylline | RO3DA | 541 |
| Glipizide | A10BB | 76 |  | Timolol | S01ED | 562 |
| Hydralazine | C02D | 140 |  | Tiotropium | R03BB | 541 |
| Indapamide | CO3D | 145 |  | Trandolapril | C09A | 161 |
| Insulins | A10A | 71 |  | Valsartan | C09C | 164 |
| Ipratoprium bromide | R03BB | 540 |  | Verapamil | C08D | 157 |
| Irbesartan | C09C | 163 |  | Vildagliptin | A10BH | 78 |
| Isardipine | C08CA | 157 |  | Warfarin | B01AA | 99 |
| Labetolol | C07A | 153 |  |  |  |  |
| Lercandipine | C08CA | 157 |  |  |  |  |
